# Supplementary material for: DNA copy number changes define spatial patterns of heterogeneity in colorectal cancer
Source: Nat Commun. 2017 Jan 25;8:14093. doi: 10.1038/ncomms14093 (PMC5288500; doi:10.1038/ncomms14093)
Supplement: Supplementary Information — Supplementary Figures and Supplementary Tables [file ncomms14093-s1.pdf]

Patient ID	Healthy tissue	Primary colon	Primary rectal	Lymph node	Liver	lung	Soft tissue	Ovary	Uterus	Skin	Brain	Retroperitoneum	Rectum	Small bowel
D002	D002N	D002P72								D002M073.1neu		D002M026		
										D002M073.2				
D003	D003N		D003P005		D003M006									
D004	D004N	D004P007			D004M008									
					D004M009									
					D004M010									
D005	D005N		D005P011.1 (mucineous)									D005M013		
			D005P011.2 (siegelring)											
D007	D007N	D007P017		D007M018				D007M019				D007M025		
D008	D008N	D008P020		D008M021			D008M022							
D010	D010N	D010P027		D010M028.1	D010M029									
				D010M028.2										
				D010M028.3										
D011	D011N	D011P030		D011M074	D011M32									
				D011M075										
D014	D014N		D014P036	D014M076										
D015	D015N	D015P038		D015M078	D015M040									
				D015M079										
				D015M080										
D016	D016N	D016P041		D016M042										
D017	D017N	D17P43-1 (block1)		D17M44	D17M81									
		D17P43-2 (block2)		D17M44.2										
		D17P43.3 (block3)		D17M44.3										
D018	D018N	D018P046			D018M082									
					D018M083									
D019	D019N	D019P048		D019M049.1	D019M050									
				D019M049.2										
D020	D020N	D020P051							D020M052			D020M053		
D021	D021N	D021P054.1 (papillary)		D021M055.2										
		D021P054.2 (adenonocarcinoma)		D021M055.5										
D022	D022N		D022P056	D022M057.1										
				D022M057.2										
D023	D023N	D023P058			D023M059									
D026	D026N	D026P064		D026M065.1								D026M066		
				D026M065.2										
				D026M065.3										
D027	D027N		D027P067	D027M068		D027M085					D027M070			
D028	D028N	D028P087		D028M088								D028M093		
D030	D030N		D030P091	D030M092						D030M094				
D032	D032		D032P099	D032M100										
D033	D033N		D033P101	D033M102	D033M104									
D034	D034N		D34P105									D34M107	D34M106	
D041	D041N		D041P124		D041M126	D041M127								D041M128
														D041M129
D050	D050N	D050P153			D050M154	D050M156						D050M157		
					D050M155									

white	no therapy
green	radio and chemotherapy
grey	other
yellow	targeted and chemotherapy
pink	chemotherapy

Supplementary Figure 1. Specification and labeling of all 27 patients and their matched metastases included in the study. P: primary tumor, M: metastasis. Multiple primary samples are a results of histological differences found in the original single tumor which prompted us to isolated the sample. Multiple primary tumors represent multiple primary samples present during original surgery or following repeated surgical excision due to re-growth of original tumor. Exposure to therapy is marked by pink for chemotherapy, green for chemotherapy with radiotherapy, yellow for targeted therapy with chemotherapy and grey for other (unknown).

a

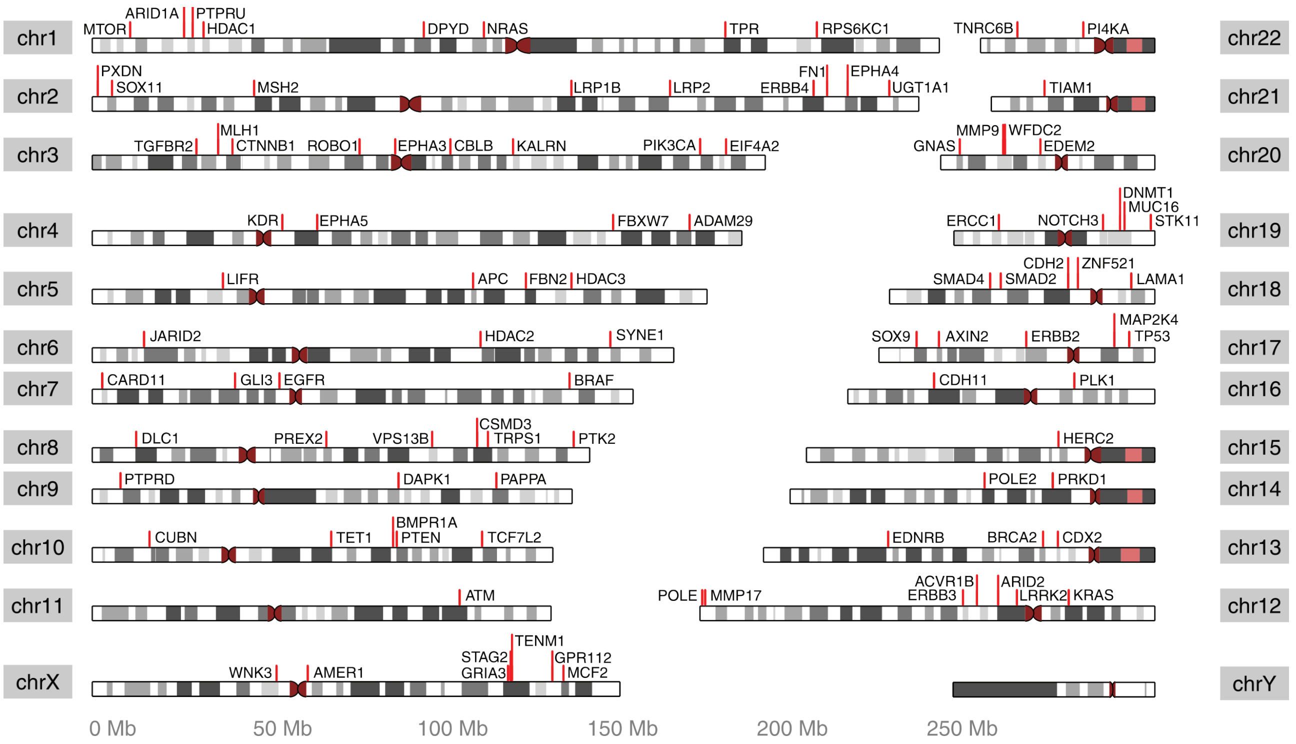

b

ACVR1B	EPHA3	LIFR	PXDN
ADAM29	EPHA4	LRP1B	ROBO1
APC	EPHA5	LRP2	SMAD2
ARID1A	ERBB2	LRRK2	SMAD4
ARID2	ERBB3	MAP2K4	SOX11
ATM	ERBB4	MCF2	SOX9
AXIN2	ERCC1	MLH1	STK11
BMPR1A	FAM123B	MMP17	SYNE1
BRAF	FBN2	MSH2	TCF7L2
CARD11	FBXW7	MTOR	TET1
CBLB	FMN2	MUC16	TGFBR2
CDH11	FN1	MUTYH	TIAM1
CDH2	GLI3	NOTCH3	TNRC6B
CDX2	GNAS	NRAS	TP53
CEP110	GPR112	ODZ1	TPR
CSMD3	GRIA3	PAPPA	TRPS1
CTNNB1	HDAC1	PI4KA	UGT1A1
CUBN	HDAC2	PIK3CA	VPS13B
DAPK1	HDAC3	PLK1	WNK3
DLC1	HERC2	POLE2	ZNF521
DNMT1	JARID2	PREX2	BRCA2
DPYD	KALRN	PRKD1	PTK2
EDNRB	KDR	PTEN	MMP9
EGFR	KRAS	PTPRD	MMP24
EIF4A2	LAMA1	PTPRU	WFDC2

c

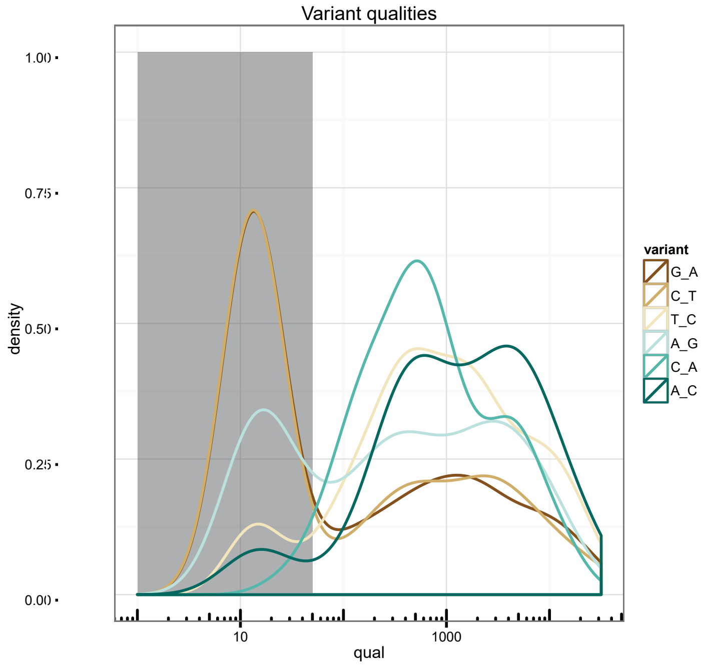

Supplementary Figure 2. CRC custom colorectal panel. (a) Overview and (b) list of genes targeted by the custom CRC panel. (c) Quality cut-off (q=50) used during the analysis to ensure minimum false positive SNVs resulting from common C > T and T > C conversions in FFPE preparations.

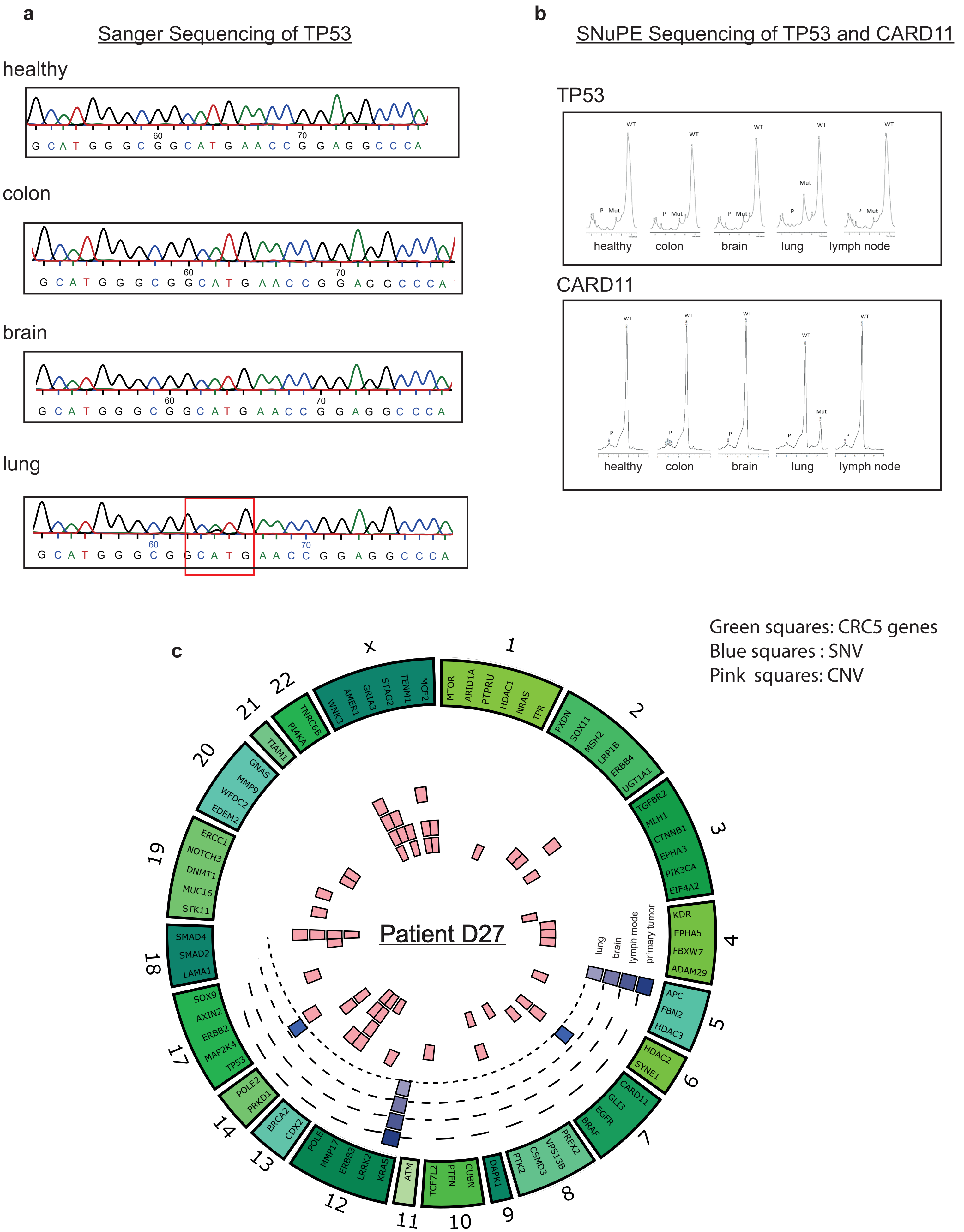

Supplementary Figure 3. Validation of SNVs in different samples from patient D27. **(a)** TP53 SNV validation by Sanger sequencing. All samples from this patient (healthy adjacent tissue, primary colon tumor, and brain and lung metastasis) were tested for the presence of the TP53 SNV. This mutation was only found in the lung sample. **(b)** SNuPE validation of TP53 and CARD11 SNVs for patient D27. **(c)** Circos diagram of patient D27 after validation. This patient displays a discordant mutation pattern private to the lung metastasis. Blue squares represent SNVs, pink squares represent CNVs (both losses and gains).

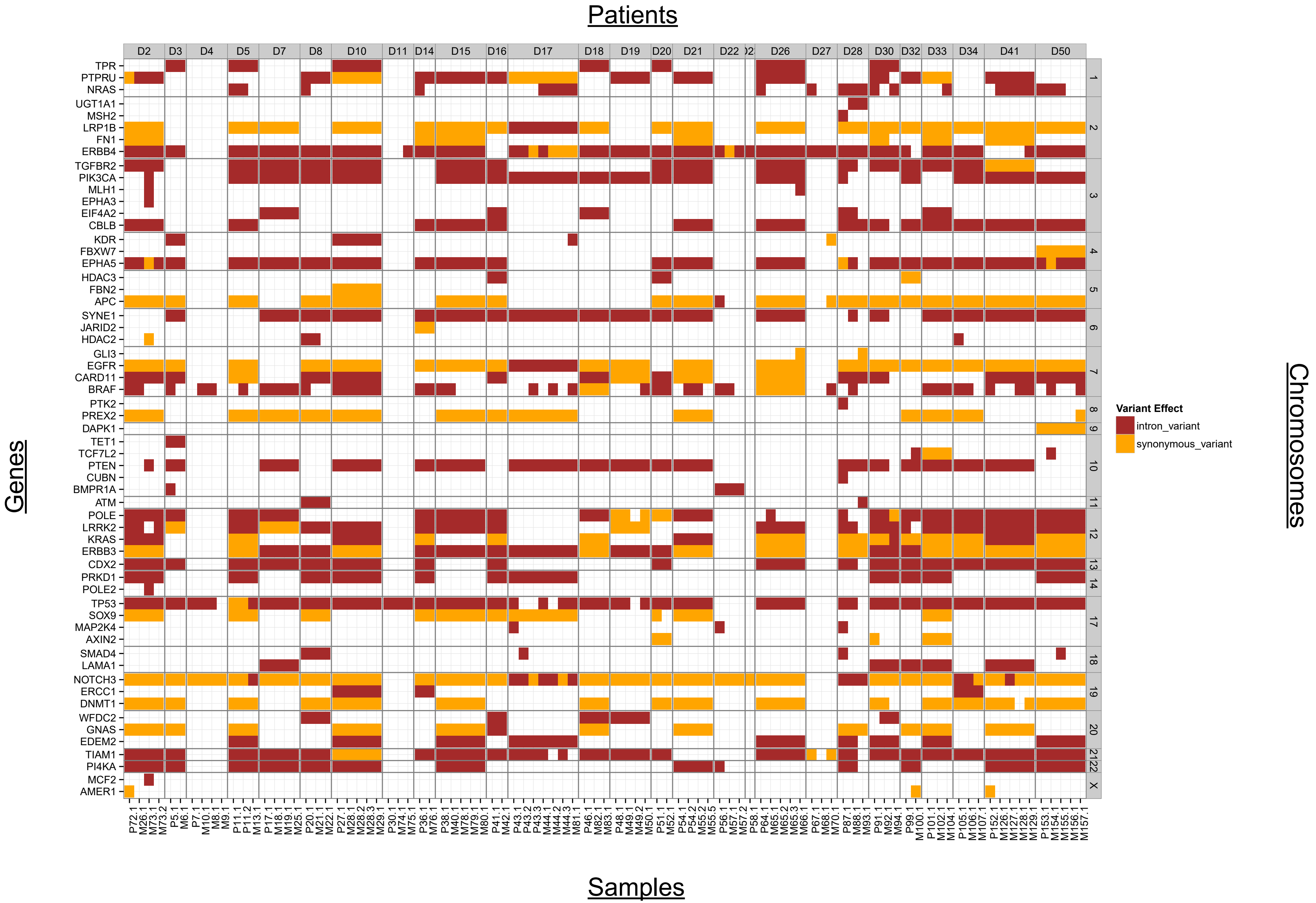

Supplementary Figure 4. Intronic and synonymous SNV profiles in matched primary and metastatic tumors identified using the CRC panel.

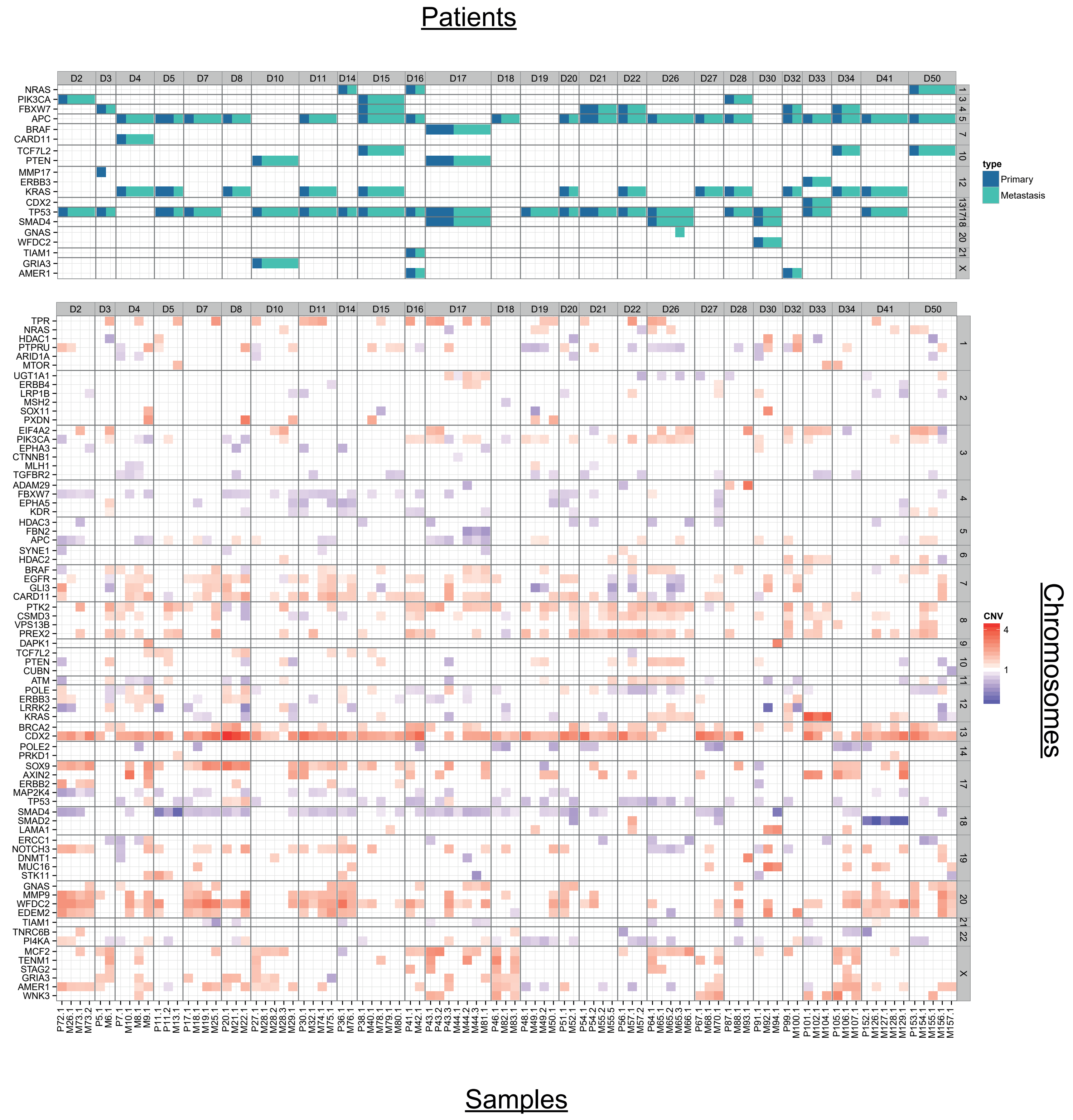

Supplementary Figure 5. Complete representation of concordant SNVs but discordant CNV profiles in matched primary and metastatic tumors. In-depth representation of alterations at the SNV level (top) and the CNV level (bottom) for each patient's matched samples. Each SNV is indicated by blue (mutation in primary tumor sample) and turquoise (mutation in metastasis). CNVs are indicated in red for gain in copy number and in blue for loss. P is primary tumor and M is metastasis.

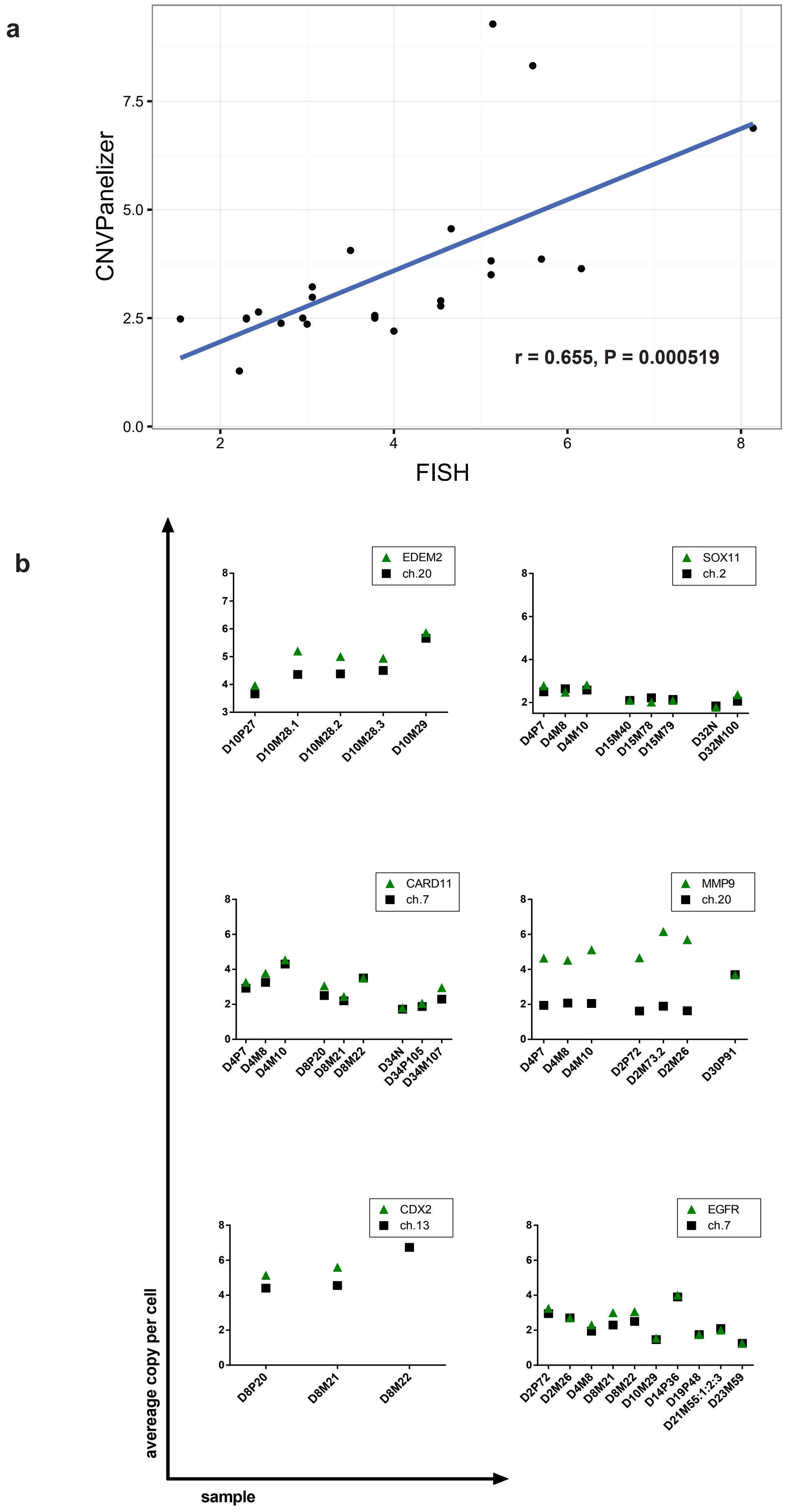

Supplementary Figure 6. (a) Comparison of CNV samples as determined by Fluorescence In-situ hybridization (FISH) (x-axis) or by CNVPanelizer from NGS data (y-axis). Resultant correlation coefficient r =0.655, p=0.000519. (only samples with 'pass' (two significant thresholds passed) were used in this analysis (refer to Oliveira et al.)). **(b)** Gene copy number counts for *EDEM2, SOX11, CARD11, MMP9, CDX2* and *EGFR* as determined by FISH using gene-specific probes (green triangles) and corresponding chromosome-specific probes (black squares). 41 samples were analyzed, 50 cells were counted from one sample, average copy number per cell are depicted here.

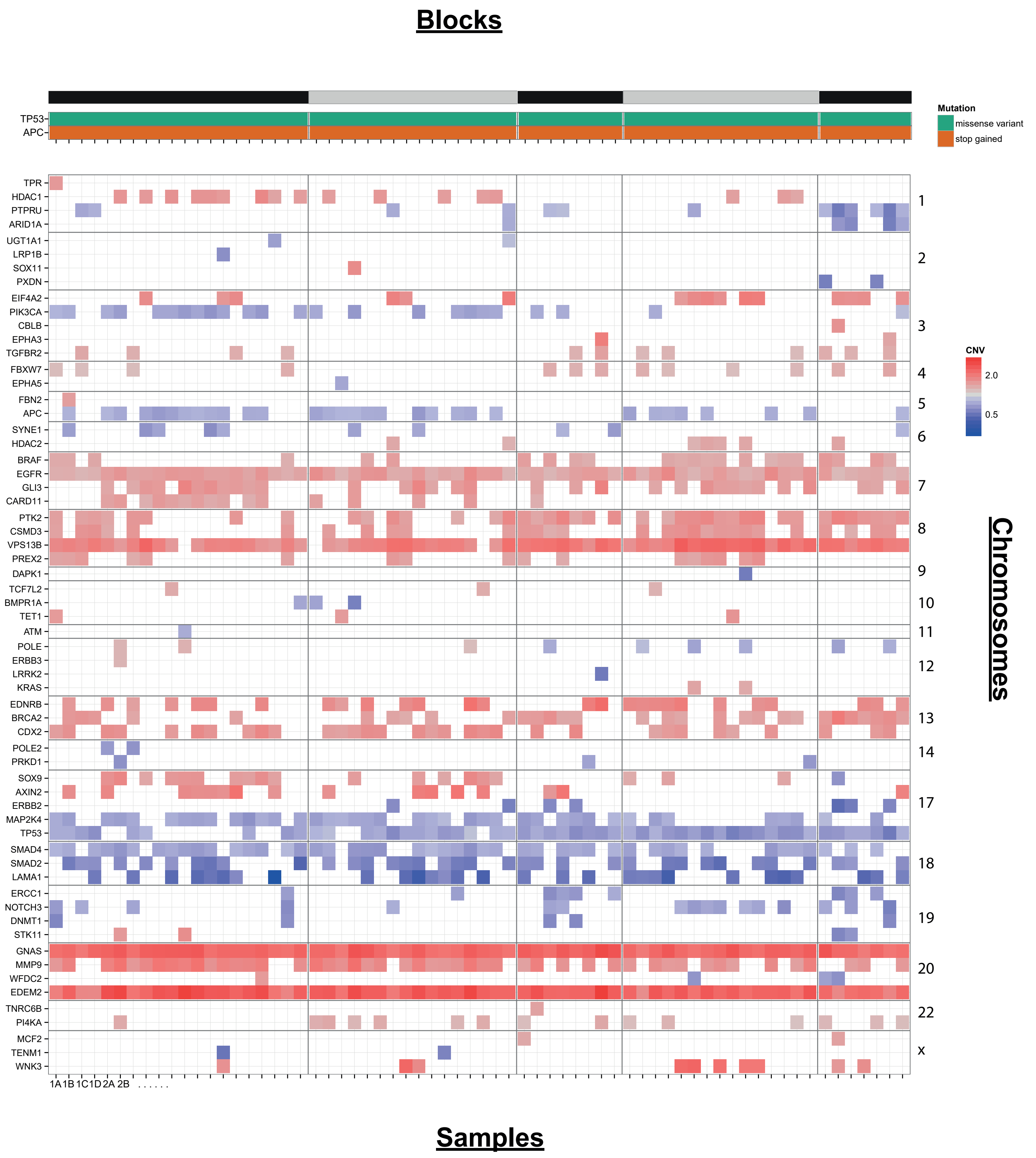

Supplementary Figure 7. Complete representation of concordant intra-tumor SNV pattern but discordant intra-tumor CNV pattern. Top panel: genetic profile of 68 samples from one CRC tumor (using the CRC panel) displays a stop-gain APC (orange) and a missense TP53 (green) alteration throughout all samples from the tumor. Lower panel: heterogeneous CNV patterns throughout the same tumor are given in blue (copy loss) and red (copy gain).

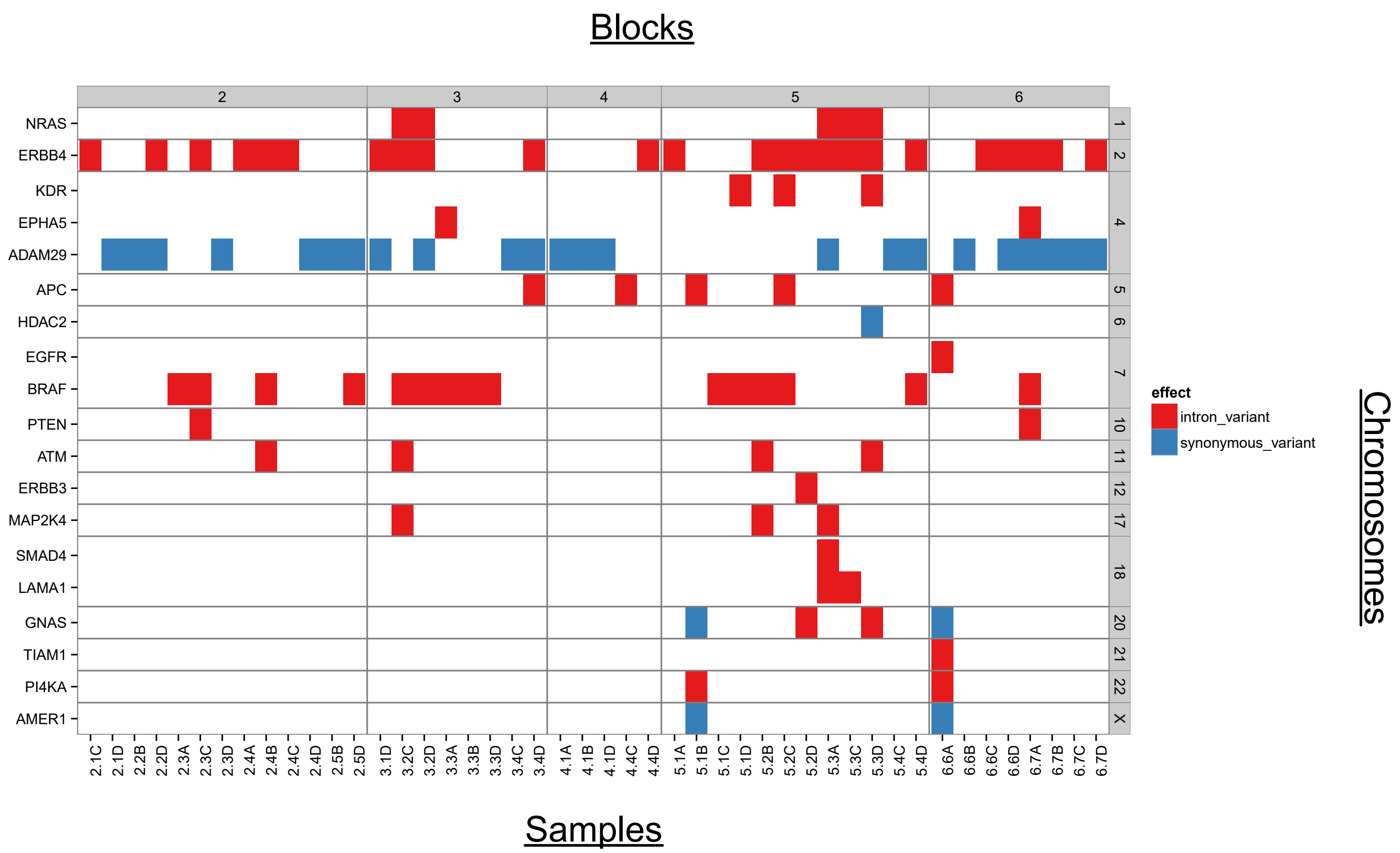

Supplementary Figure 8. Intronic and synonymous SNV profile in 68 samples from one CRC tumor identified using the CRC panel.

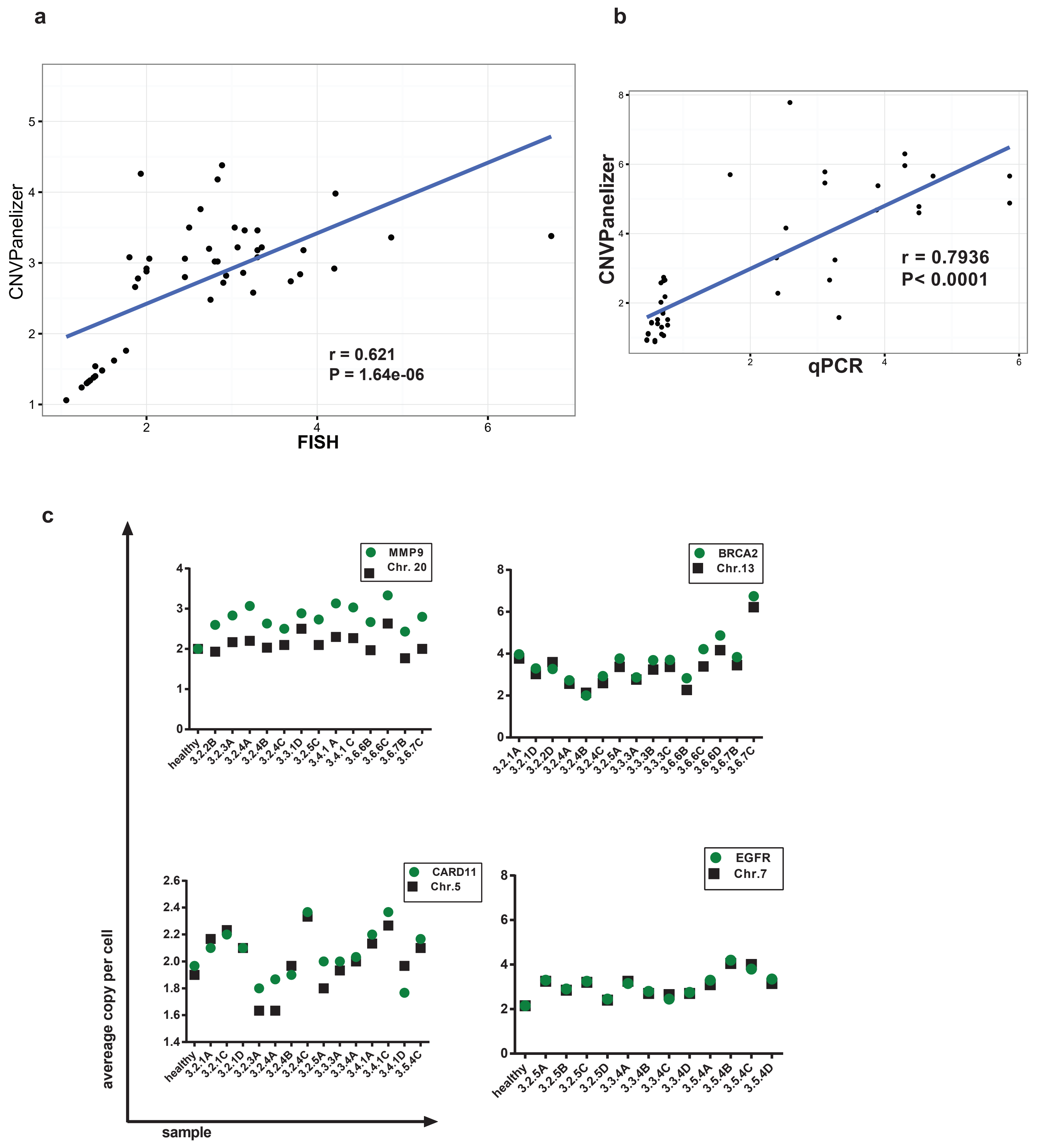

Supplementary Figure 9. (a) FISH analysis of E*GFR, TP53, BRCA2, MMP9* and *CARD11* in a subset of the 68 samples derived from the single tumor resultant correlation coefficient shows r = 0.621 (only samples with 'pass' (two significant thresholds passed) were used in this analysis (refer to Oliveira et al.)) **(b)** qPCR validation of CNVs from *APC, EDEM2, GNAS, HDAC3, MAP2K4, MMP9, P53, SMAD4* and *SOX9* genes. The TERT gene served as a reference. Data was compared to CNVPanelizer output, resulting with correlation coefficient of r = 0.7936. **(c)** Gene copy number counts for *MMP9, BRCA2, CARD11* and *EGFR* as determined by FISH using gene-specific probes (green circles) and corresponding chromosome-specific probes (black squares). 73 samples were analyzed, 30 cells were counted from one sample, average copy number per cell is depicted here.

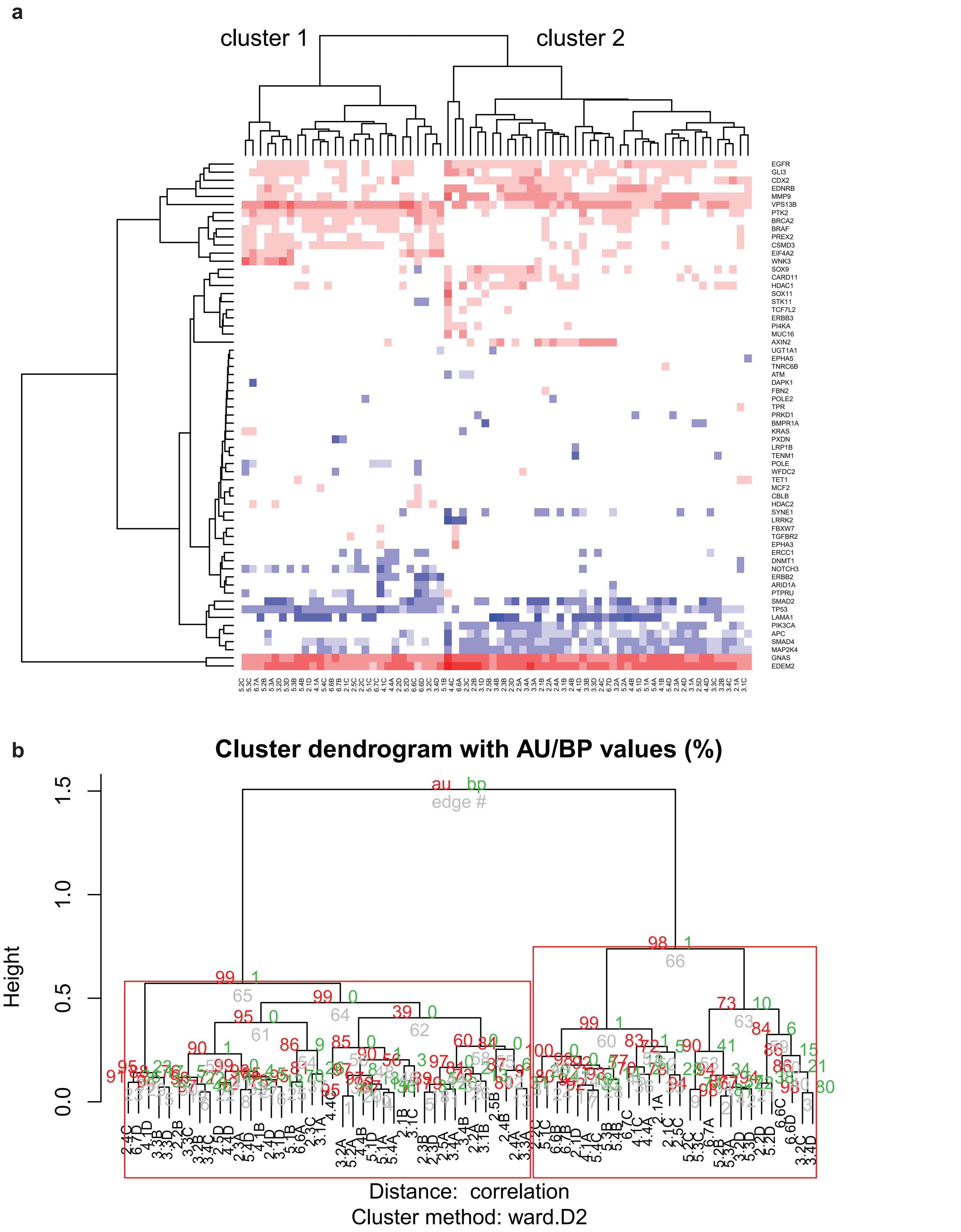

Supplementary Figure 10: (a) Clustering of CNVs derived by CNVPanelizer from the 68 samples within the tumor using agglomerative hierarchical clustering. The objective function used for clustering was the root sum of squared distances between sub-cluster points; the values of each point were the predicted copy number of each gene represented by the panel. Samples clustered into two distinct clusters; cluster 1 and cluster 2. **(b)** The stability of the clusters was assessed by bootstrapping tree construction with 10,000 repetitions and calculating the approximate unbiased p-value (AU). The two main clusters have an AU of 99 indicating that they are highly reliable.

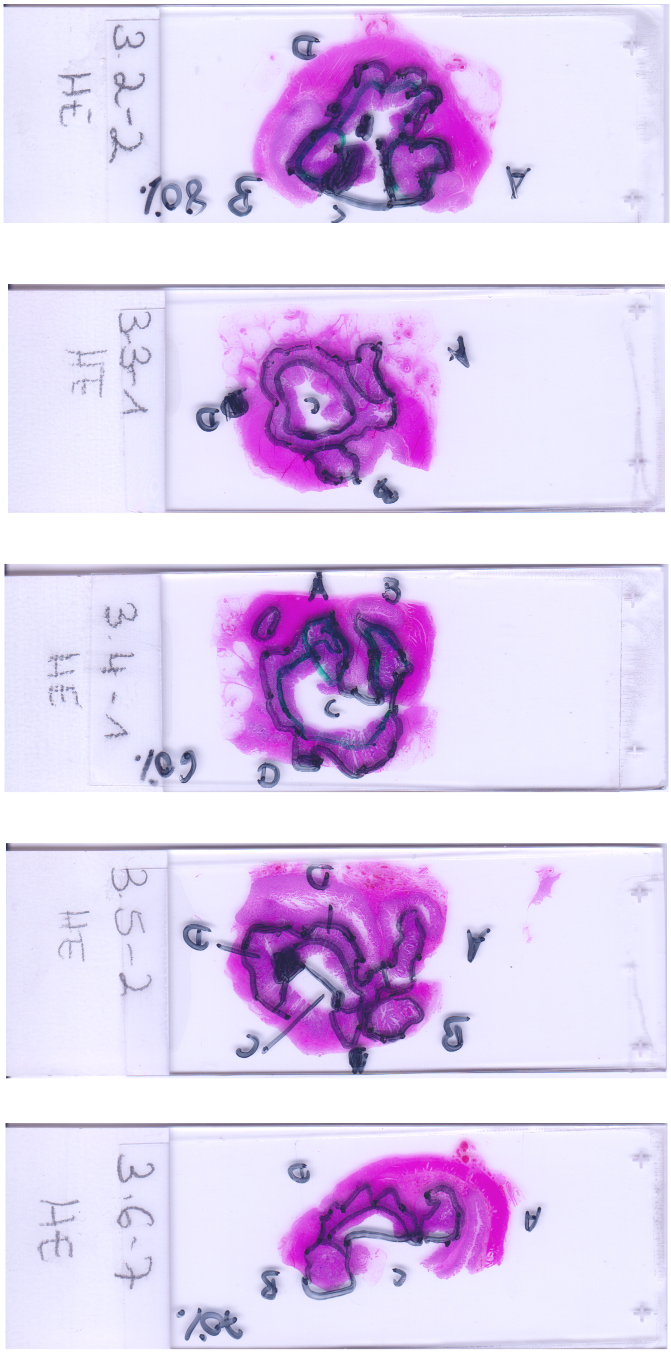

Supplementary Figure 11. Representative macro images of tissue H&E sections used for the 3D reconstruction model. Areas designated A, B, C and D correspond to the left and right lateral tumor regions, and the luminal and deep invasive front, respectively.

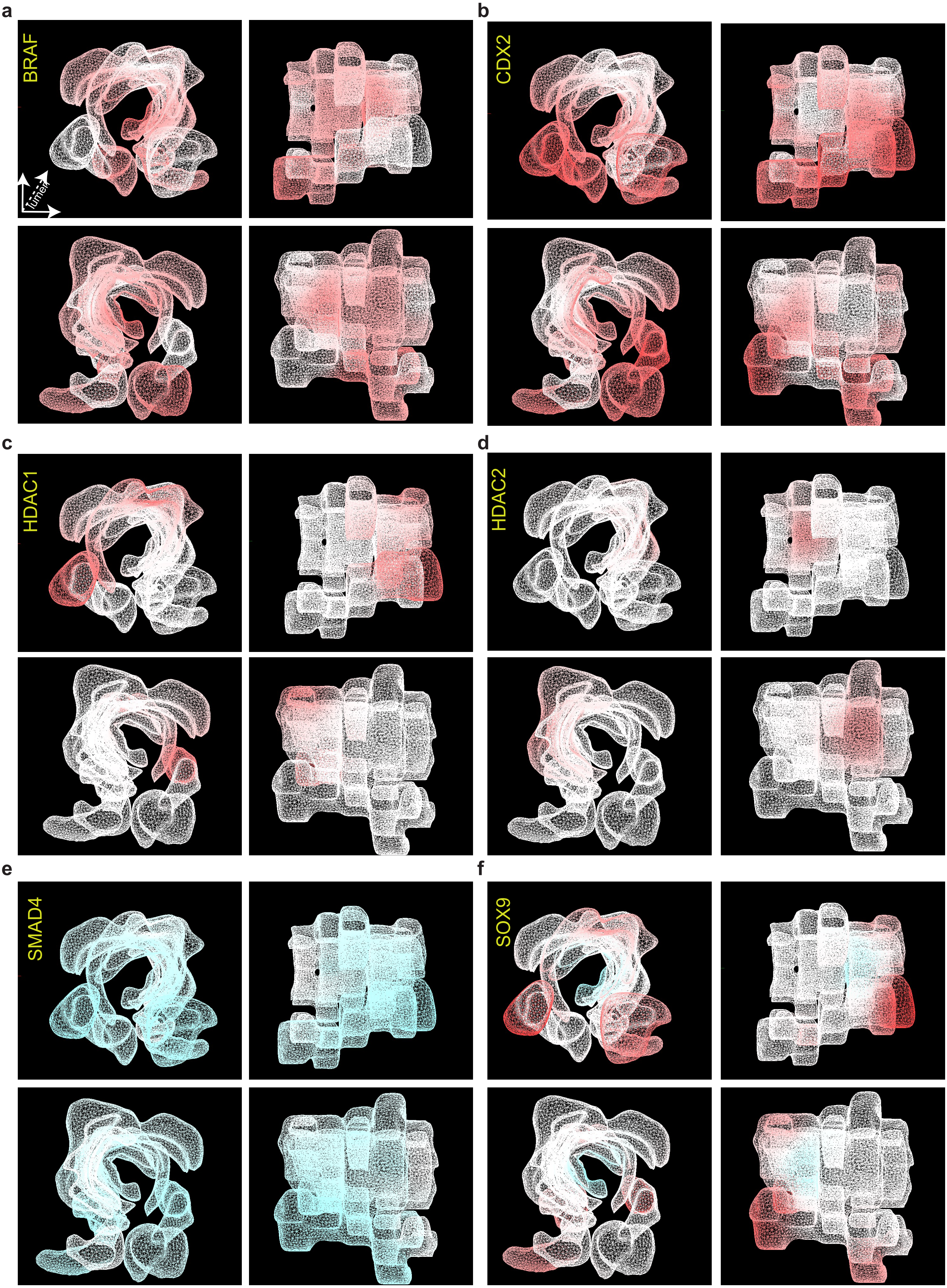

Supplementary Figure 12. 3D morphological tumor models generated from slides stained with H&E, overlaid with distribution of CNVs from the 68 samples investigated representing (b) *BRAF*, (c) *CDX2*, (b) *HDAC1*, (d) *HDAC2*, (e) *SMAD4* and (f) *SOX9.*

Supplementary Table 1. Samples found in cluster 1 and cluster 2.

sample	cluster
3.3.2C	1
3.2.1D	1
3.3.4D	1
3.2.1C	1
3.5.3C	1
3.2.5C	1
3.5.3D	1
3.5.2C	1
3.5.1C	1
3.4.1C	1
3.6.7C	1
3.6.6D	1
3.6.6C	1
3.6.7B	1
3.6.6B	1
3.4.1A	1
3.5.4C	1
3.2.2C	1
3.5.3A	1
3.5.2D	1
3.3.2D	1
3.6.7A	1
3.5.2B	1
3.2.1A	1
3.4.4A	1
3.5.4B	2
3.4.1D	1
3.5.3B	1
3.6.7D	2
3.5.2A	1
3.2.2D	2
3.4.4D	2
3.3.2A	2
3.4.4C	1
3.2.4C	2
3.4.4B	2
3.2.5B	2
3.4.1B	2
3.3.1C	2
3.5.1B	2
3.3.3D	2
3.2.1B	2
3.3.3B	2
3.6.6A	2
3.2.4B	2
3.5.4D	2
3.3.3C	2
3.2.5D	2
3.5.1A	2
3.3.3A	2
3.5.4A	2
3.3.1A	2
3.3.4C	2
3.3.1D	2
3.2.3C	2
3.3.2B	2
3.3.4B	2
3.2.3A	2
3.3.4A	2
3.2.2B	2
3.3.1B	2
3.2.2A	2
3.2.4A	2
3.2.5A	2
3.5.1D	2
3.2.4D	2
3.2.3D	2
3.2.3B	2

Supplementary Table 2. p values of CNV differences between the clusters. Key genes are shown.

Gene	P value
CSMD3	7.49E-08
PTK2	3.33E-06
BRCA2	1.86E-05
NOTCH3	1.94E-05
MMP9	6.81E-05
PREX2	1.38E-04
BRAF	2.32E-04
CARD11	2.54E-04
HDAC1	6.01E-03

Supplementary Table 3. Primers used for Sanger sequencing.

Inter-tumor heterogeneity

Gene	Position	Forward primer sequence	Reverse primer sequence
APC	5:112174724	CGAGTGGGTTCTAATCATGGA	AGGCTGATCCACATGACGTT
SMAD4	18: 48,591,825	AGCTCCTGAGTATTGGTGTTCC	TCAATGGCTTCTGTCCTGTG
PIK3CA	3: 178,937,373	TGTATTGCTTGGTAAAAGATTGG	GCTGAATTAAATACTGAGAAAGTTTG
FBXW7	4: 153244227	CCCACTCTCCAATGTGACTAGGT	TGGTTTTTGTCTAGGTCCCAACAA
CARD11	7:2984030	TGAACGAGGTCATCAAGCTG	CCTCACTGAGCTGTGCGTAG
PIK3CA	3: 178947179	CCTTATGGTTGTCTGTCAATCGGT	TACAACTCACATTTCTCCTTTGTTCTTGT
NOTCH3	19: 15285135	AGTACTGCGCCGACCACTT	GAACGCAGTAGCTCCTCTGG
ARID1A	1:27058024	CTCCCAGCAGTCGACGACAC	TAGGCAGTTTGCTGGGACTG
FBXW7	4:153245446	AAAGGTGAGTAAGACTTACCTTGCA	ATGTGGAGACAGGGAATTGCAT
FBXW7	4:153247367	ACACCTTTACCATAAAATCATATGCTCCA	GTTTCTCCCTCTGCAGAGTTGT
TCF7L2	10: 114901021	CGGTGTCTTTCTCTGTTCTCCT	CCCACAGCCTACCTGTTTTGG
SMAD4	18:48591919	CTGAGTATTGGTGTTCCATTGCTTAC	CTCTCAATGGCTTCTGTCCTGT
TP53	17:7577545	GGCTCCTGACCTGGAGTCTT	CTCATCTTGGGCCTGTGTTATCTC
AMER1	x:63412095	CCTCGTCATCATCATCTGGCAA	TTCATTGACAGGTTGTGGTGACA
DLC1	8:13356821	CAGTCCTCTGTTTGTGCAAGGA	TGCAGTGGATACTTTGAACGTGAA
ERBB3	12:56481620	CCCTAACAGCCATGCTTTCTCT	GGAAAGGGTACATACAAAGCAGTCT
CDX2	13:28537449	CTCTGGGACACTTCTCAGAGGA	TCCACCTTTCCATTTCTAGGTTAAAATCTG
TIAM1	21:32624093	CTCCCGCTCTCACCTTTCAG	CAGTCGGACATCCTGCTGAC
AMER1	x: 63411365	GCATAGGCTTCCCTGCCATAAG	CTAGTGACCATCCAGAAACAGTTGT
SMAD4	18:48604750	TGTTGATGACCTTCGTCGCTTA	TCTGCAATCGGCATGGTATGAA
DNMT1	19:10267076	CCCAAACATAATCCCGGACTATTCC	TAAGCATGTGCTTTGTTTCCTGTCTA
TIAM1	21: 32624166	CTCCCGCTCTCACCTTTCAG	CAGTCGGACATCCTGCTGAC
SMAD4	18:48604787	TGTTGATGACCTTCGTCGCTTA	TCTGCAATCGGCATGGTATGAA
ERBB4	2:212652762	TAAGCATAACTCATTCATCGCCACAT	GCAGACACCATTCATTGGCAAG
APC	5:112173887	CATCAAGAGGAAGCTTAGATAGTTCTCG	CTTCTTCCATGACTTTGGCAATCTG
PTEN	10:89711910	CCAGGGAGTAACTATTCCCAGTCA	AGAATTTCAAGCACTTACTGCAAGTTC
DNMT1	19:10267076	CCCAAACATAATCCCGGACTATTCC	TAAGCATGTGCTTTGTTTCCTGTCTA
TIAM1	21:32624166	CTCCCGCTCTCACCTTTCAG	CAGTCGGACATCCTGCTGAC
ARID1A	1:27105929	AGTAAGTTTGACAAGCTTCCAGTAAAGA	GCTCTGTCTTGCTCTCGAAGT
AXIN2	17:63532566	TCGACACCTCAGCTAGCCT	TCGTCTCCAGGCGAACGAG
PIK3CA	3:178952076	GGCTCTGGAATGCCAGAACTAC	GTTTAATTGTGTGGAAGATCCAATCCATTT
APC	5:112174572	CTGAGAGCACTGATGATAAACACCT	TCTTCATAGTCATCTTCTTGACACAAAGAC
ATM	11:108186638	TTCCTTCTTCAATTTTTGTTGTTTCCATGT	TCCTTCATAAACAGAATTACTATACCGTTGTTT
PIK3CA	3:178936082	ATTTTACAGAGTAACAGACTAGCTAGAGACA	CTCCATTTTAGCACTTACCTGTGACT
SMAD4	18:48591918	CTGAGTATTGGTGTTCCATTGCTTAC	CTCTCAATGGCTTCTGTCCTGT

PIK3CA	3:178936091	ATTTTACAGAGTAACAGACTAGCTAGAGACA	CTCCATTTTAGCACTTACCTGTGACT
KRAS	12:25378647	GCCTGTTTTGTGTCTACTGTTCTAGA	GACAAAAGTTGTGGACAGGTTTTGA
PTEN	10:89692892	TGAGGTTATCTTTTTACCACAGTTGCA	ATATCATTACACCAGTTCGTCCCTTTC
FBXW7	4:153258954	TGACAATGTTTAAAGGTGGTAGCTGT	TCATTGATAGTTGTGAACCAACACAAGT
APC	5:112175473	GACACCCAAAAGTCCACCTGAA	GCTTATAATGCCACTTACCATTCCACT
TP53	17:7577142	CTTGCTTACCTCGCTTAGTGCT	CTTGCTTCTCTTTTCCTATCCTGAGT
ARID1A	1:27057934	CTCCCAGCAGTCGACGACAC	TAGGCAGTTTGCTGGGACTG
SMAD4	18:48604787	TGTTGATGACCTTCGTCGCTTA	TCTGCAATCGGCATGGTATGAA
KRAS	12:25378648	GCCTGTTTTGTGTCTACTGTTCTAGA	GACAAAAGTTGTGGACAGGTTTTGA

Intra-tumor heterogeneity

Gene	Position	Forward primer sequence	Reverse primer sequence
APC	5:112175390	AGAATCAGCCAGGCACAAAG	GCAATCGAACGACTCTCAAA
TP53	17:7578406	CAGCTGCTCACCATCGCTAT	GTTTTGCCAACTGGCCAAGA
APC	5:112175951	CCAGTTCAGGAAAATGACAATGGGAA	GGCATGGCAGAAATAATACATTCTTCTAGT
PIK3CA	3: 178952103	TGAGCAAGAGGCTTTGGAGT	ATGCTGTTCATGGATTGTGC
BRAF	7:140501343	TTCTGTACTACAACGCTGGTGAAA	GTCAGTTTCTAGAAAGTTTTCTTGTGAGTT
ERBB4	2:212248464	CTTCAGGGAGAACTCAGAGAGGTA	ACAACCCTGACTACTGGAACCA
MCF2	x:138697051	TGAAGAATAAACTCAGTATTTACATCAAGAGT	AATTTGTGGTACCCACACCTGAAA
WNK3	x:54276057	TCGTCAGTACGTGCTTAGCC	CCATCAACCACATCCTTGTGT
APC	5:112173729	TGACACCAATCGACATGATGATAATAGG	AATTCCGCGTTCTCTCTCCAAA

Supplementary Table 4. Primers used for SnuPE sequencing.

Inter-tumor heterogeneity

locus	SNuPE primer 5´-3´´
MMP17	tggagcctggcgt
MUC16	agagatgtcaccc
LRP2	gcacaggcgcag
GRIA3	ctcctcaggtag
ARID1A	gcaccccagagc
NOTCH3	tctcacatagtg
HDAC2	gctcctcctcct
KRas	cagcaggtca
KRas	ttgaaacatcag
CARD11	atcgagatggtg
BMPR1A	ctggtcatcttct
GNAS	gaagtcaggaca
APC	caagtcatctgg
APC	gtcatctgggaa
TP53	aggggccacgg
TP53	tgggagcttcat
NRAS	acaaggacagtt
ERBB4	tacctgcaggag
CTNNB1	aagccctcacga
EPHA3	ttggtgtgtaag
LIFR	ttgtttttatt
LRRK2	aaatcattttat
PIK3CA	agaacccagaca
TIAM1	aagcaagctgccc
BRAF	gtaaaaataggtg
DLC1	tgcaatgtagta
PIK3CA	gaaatcactgag
FBXW7	ccacttgttaac
FBXW7	gctccctaaaga
TCF7L2	cttgtatgtagc
AMER1	tcactttgctca
FBXW7	cctcctgccat
TGFBR2	gactgagtgctg
POLE	ctgatgctgaga
EGFR	tcaactcccaaa
TCF7L2	agcaatgaacac
FBXW7	gagtggcatctc
APC	tcaactgctcat
KDR	ccaccagagatt
KDR	atccttggtcac

APC	tccaacttctc
APC	tcgaacgactct
POLE	accgaggaaatg
TP53_0894f	GGCTCCTGACCTGGAGTCTT
TP53_0894r	CTCATCTTGGGCCTGTGTTATCTC

Intra-tumor heterogeneity

**locus**	**SNuPE primer 5´-3´´**
TP53_3739	aggttgtgaggc
APC_1391	caaaagtggtgct
NOTCH3_8924	acggaggagtgc
ADAM29_8779	caactgaggttg
MCF2_9052 top strand	ccttgtttagat
MCF2_9052 bottom strand	tgggacaccatttttt

Supplementary Table 5. Primers used for qPCR.

Gene	Primer name	Forward primer sequence	Reverse primer sequence	samples tested
Tp53	Tp53.1	GGCCCACCTCTTACCGATTT	CAGTAAGGAGATTCCCCGCC	3.2.2B, 3.3.4C, 3.4.4D, 3.6.7C
Tp53	Tp53.2	CTCTGTTGCTGCAGATCCGT	GCCTCATTCAGCTCTCGGAA	
MAP2K4	MAP2K4.1	GGGGAAGACTTGCCTGCTAC	GCCTCCTACCACGCAAAATG	3.2.2B, 3.3.4C, 3.4.4D, 3.6.7C
MAP2K4	MAP2K4.2	CCCACACATGTGAGTATTCTTGG	ATCTTTGCTTGAGTTGAGCCTTT	
MMP9	MMP9.1	CCGCTATGGTTACACTCGGG	GGCCCCAGAGATTTCGACTC	3.2.3A, 3.2.5C, 3.4.4A, 3.5.4D
MMP9	MMP9.2	GCAGACCATCCATGGGTCAA	AAGGCGGAGACAGCACATTA	
GNAS	GNAS.1	CCAGGCCAGTGATGTCATGT	ATCTCATCCCTCTGCTGGGT	3.2.3A, 3.2.5C, 3.4.4A, 3.4.4C
GNAS	GNAS.2	CCCTACACTCCAGCACACAG	GGCCGAGTTGGGATGGATAG	
EDEM2	EDEM2.1	TGAATTTGCCACCCTGAGCA	TGAATTTGCCACCCTGAGCA	3.2.3D, 3.3.4A, 3.5.1D, 3.5.4C
EDEM2	EDEM2.2	CAGGACAGCGTGGACTTTGA	GCAGCCCTTACCTCGAATGT	
SOX9	SOX9.1	CAAAGTTCTTGGGCTGCTCG	CAGCTACCTCCCTCACCTCT	3.2.3A, 3.2.5C, 3.4.4A, 3.5.4D
SOX9	SOX9.2	AGCTTTGGTTTGTGTTCGTGT	GCTGGGAGGGAAACAAGTGA	
APC	APC.1	TCCCTGGAGTAAAACTGCGG	CCGGCTTCCATAAGAACGGA	3.2.2D, 3.4.4B, 3.5.1A, 3.5.4C
APC	APC.2	TTGGCCGAGTCTTGTAGTGC	GCCTGCTAACATTCTGCGTT	
SMAD4	SMAD4.1	CTTTGAGGGACAGCCATCGT	CTTGGTGGATGCTGGATGGT	3.2.2B, 3.3.4C, 3.4.4D, 3.6.7C
SMAD4	SMAD4.2	GTTGTCCAGTGCATTGCAGG	TGGGCATCTGGTGGCTAAAG	
HDAC3	HDAC3.1	AGATGATCGTGAGTTCCGCC	TCCAGCCCACCTATCCCTAC	3.2.3D, 3.5.1B, 3.5.4C, 3.6.6A
HDAC3	HDAC3.2	ACCAGATCCGCCAGACAATC	TGCAGGCACGTCATGAATCT	
TERT	TERT_1	CCTACCGTGGCAGCTAGAAG	CTGCATGGCCGGAAGTCTTA	reference primer
TERT	TERT_2	GGTTGTGGTGACGTTGCTTC	CCAGGTTTGCAAGCTGACAC	reference primer
RPPH1	RPPH1_1	GGAGCTCAATATCGCGGGAC	GTCACAGTAGGTGGCATCGT	reference primer
RPPH1	RPPH1_2	CAGGAACCGTAGGCTTTCGT	TGGTGGTTTCCAACTGCGAT	reference primer

Supplementary Table 6. Overview of SNV validation results for intra-tumor heterogeneity study. Several SNVs were not found at sufficient depth to be 'called' by the variant caller pipeline, we therefore validated their presence or absence using Sanger Sequencing (primer sequences can be found in table 3).

Gene	Position	Sample	Called	Result from Sanger
**APC**	chr:5_112175390	3.1.1 (healthy)	no	not present
		3.2.1C	yes	present
		3.3.1A	yes	present
		3.4.4B	yes	present
		3.5.1D	yes	present
		3.6.6B	yes	present
		3.2.3C	**no**	present
		3.3.4B	**no**	present
		3.5.3D	**no**	present
		3.5.4B	**no**	present
		3.6.7D	**no**	present
**TP53**	chr:17_7578406	3.1.1 (healthy)	no	not present
		3.3.2C	yes	present
		3.3.3A	yes	present
		3.5.1B	yes	present
**APC**	chr:5_112175951	3.1.1 (healthy)	no	not present
		3.3.2C	**yes**	not present
		3.2.4A	no	not present
		3.5.2C	no	not present
		3.4.4B	no	not present
		3.5.1A	no	not present
		3.6.6C	no	not present
		3.6.6D	no	not present
**PIK3CA**	chr:3_ 178952103	3.1.1 (healthy)	no	not present
		3.2.4C	no	not present
		3.2.5A	no	not present
		3.2.5B	no	not present
		3.3.1D	no	not present
		3.3.2D	**yes**	not present
		3.5.1B	no	not present
**BRAF**	chr:7_140501343	3.1.1 (healthy)	no	not present
		3.2.1A	no	not present
		3.3.1C	no	not present
		3.4.4A	no	not present
		3.5.2C	no	not present

**ERBB4**	chr:2_212248464	3.1.1 (healthy)	no	not present
		3.4.4C	no	not present
		3.5.2D	no	not present
		3.5.3A	**yes**	not present
		3.6.7C	no	not present
		3.5.3C	**yes**	not present
		3.6.6C	**yes**	not present
		3.6.6D	**yes**	not present
**MCF2**	chr:X_138697051	3.1.1 (healthy)	no	not present
		3.5.3A	**yes**	not present
**WNK3**	chr:X_54276057	3.1.1 (healthy)	no	not present
		3.3.3A	no	not present
		3.3.4B	no	not present
		3.5.1A	no	not present
		3.5.2C	no	not present
		3.5.3A	**yes**	not present
**APC**	chr:5_112173729	3.1.1 (healthy)	no	not present
		3.5.3D	yes	not present

Supplementary Table 7. Data used for the visualization of the screenshots in Fig. 6b - d. Each table contains measured data of copy number variation for one gene from our experiments. Columns correlate to regions A to D and rows correspond to the slice planes.

clusters	A	B	C	D
Plane 1	2	2	1	2
Plane 2	2	2	2	2
Plane 3	1	2	1	1
Plane 4	1	1	1	1
Plane 5	1	1	1	2

BRCA2	A	B	C	D
Plane 1	1	1	1.38	1
Plane 2	1	1	1	2.94
Plane 3	1.49	1.5	1.66	1.36
Plane 4	1.45	1.37	1	1
Plane 5	1.47	1.59	1.69	1.45

Card11	A	B	C	D
Plane 1	1.42	1.51	1	1.29
Plane 2	1.43	1	1	1.51
Plane 3	1	1.29	1	1
Plane 4	1	1	1	1
Plane 5	1	1	1	1

MMP9	A	B	C	D
Plane 1	1.87	2.13	1.55	1.75
Plane 2	2.04	1.77	1.65	2.19
Plane 3	1.43	1.9	1	1.6
Plane 4	1.5	1.54	1	1.54
Plane 5	1.56	1	1	1.54
