# Supplementary figures and images for: DNA copy number changes define spatial patterns of heterogeneity in colorectal cancer
Source: Nat Commun. 2017 Jan 25;8:14093. doi: 10.1038/ncomms14093 (PMC5288500; doi:10.1038/ncomms14093)

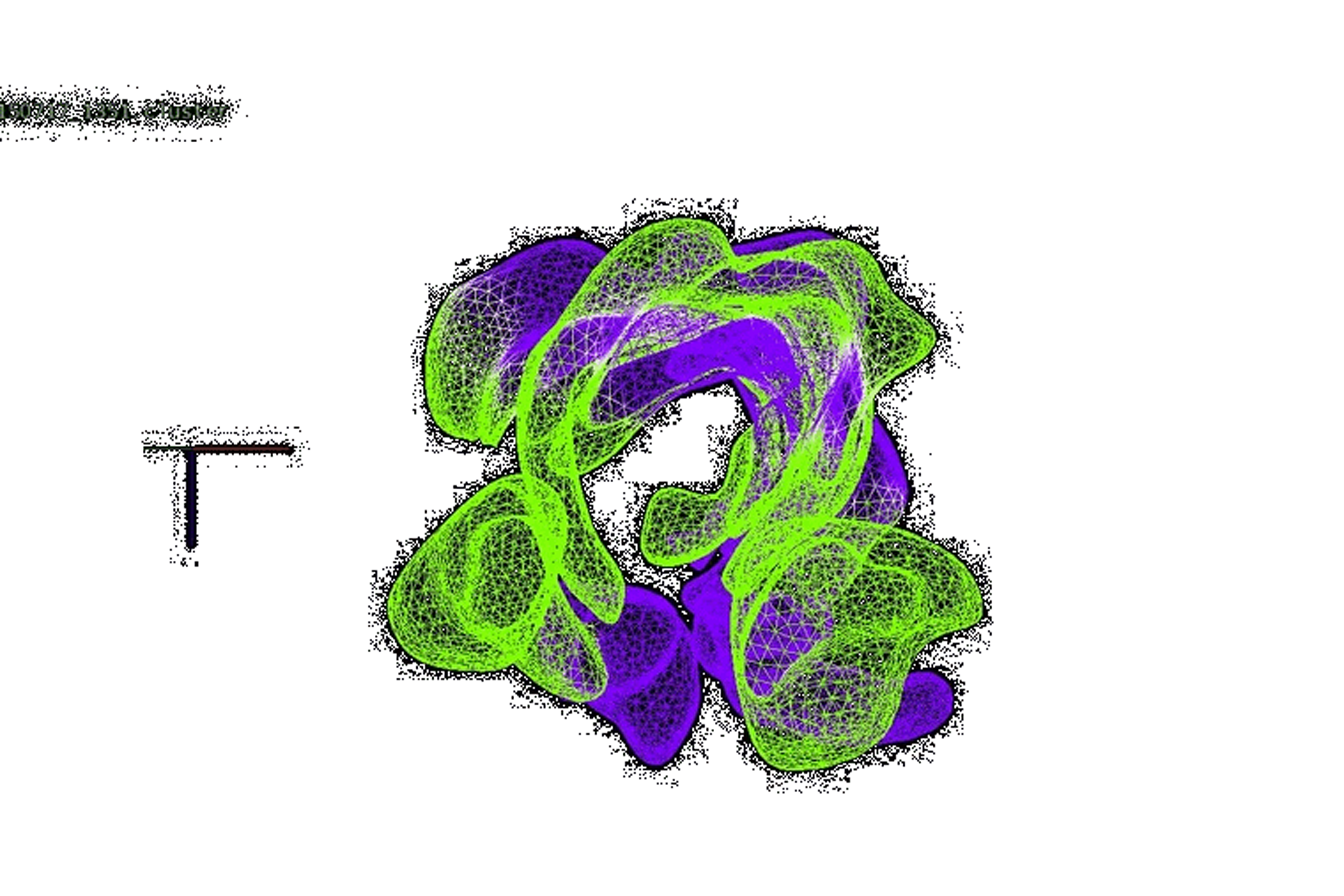

Supplement: Supplementary Movie 1 — 3D reconstruction of a stage II colon cancer rotating 360° around the horizontal axis and color coded by DNA copy number variation (CNV). Two main clusters are shown in green and violet. Red, blue and green axis key (left) indicates the x, y and z axis respectively. Z axis denominates the proximal - distal axis along the colon lumen. Distinct compartments are removed per rotation to expose luminal structures. Rotation 1 shows complete tumor. Rotations 2 and 3 expose the luminal compartment by removal of the right and left lateral compartments, respectively. In rotation 4 the deep invasive front is eliminated, revealing the luminal invasive front compartment. [file ncomms14093-s4.tif]
